# Peripheral inflammation in prodromal Alzheimer’s and Lewy body dementias

**DOI:** 10.1136/jnnp-2017-317134

**Published:** 2017-12-16

**Authors:** Eleanor King, John Tiernan O’Brien, Paul Donaghy, Christopher Morris, Nicola Barnett, Kirsty Olsen, Carmen Martin-Ruiz, John-Paul Taylor, Alan J Thomas

**Affiliations:** Institute of Neuroscience, Campus for Ageing and Vitality, Newcastle University, Newcastle upon Tyne, UK

## Abstract

**Objectives:**

There is growing evidence for the role of systemic inflammation in Alzheimer’s disease (AD) and other neurodegenerative diseases; however the systemic inflammatory profile in dementia with Lewy bodies (DLB) has never before been investigated. This study aimed to characterise systemic inflammatory mediators in established DLB and AD, as well as in their prodromal, mild cognitive impairment (MCI) phases.

**Methods:**

We obtained plasma samples from patients with DLB (n=37), AD (n=20), MCI with DLB profile (n=38), MCI with AD profile (n=20) and healthy control subjects (n=20). The following inflammatory biomarkers were measured using Roche cobas c702 and Meso Scale Discovery V-Plex Plus: high-sensitivity C-reactive protein, interferon-gamma, interleukin (IL)-10, IL-12p70, IL-13, IL-1beta, IL-2, IL-4, IL-6, IL-8 and tumour necrosis factor-alpha.

**Results:**

We found significantly higher levels of IL-10, IL-1beta, IL-4 and IL-2 in both MCI groups (P<0.001), while there was no significant difference in inflammatory markers between dementia groups and controls. Furthermore, increased disease severity was associated with lower levels of IL-1beta, IL-2 and IL-4 (P<0.05).

**Interpretation:**

We have shown for the first time that in both DLB and AD, increased peripheral inflammation occurs early at the MCI disease stages. These data support a role for inflammation early in the disease process, and have important implications for the stage of disease where trials of anti-inflammatory medication should be focused.

## Introduction

Dementia affects around 50 million people worldwide, and this is expected to rise to 131.5 million by 2050. One of the major problems in dementia care is the lack of effective treatments, with current pharmacological therapies having limited benefit.

Theories about the role of neuroinflammation in the progression of degenerative dementia have been gaining interest due to their therapeutic potential. It has been suggested that neuronal damage in chronic neurodegeneration leads to a damaging proinflammatory microglial response.^1^ In Alzheimer’s disease (AD) there has been considerable evidence to show that peripheral and central inflammations play a key role in the pathogenesis of the disease. While serum tumour necrosis factor (TNF)-alpha in particular appears to be highly associated with cognitive decline,^2^ associations have been made with several other systemic inflammatory markers.^3^ Longitudinal studies have found that inflammation occurs years before AD onset,^4 5^ and cross-sectional studies have found a large increase in inflammatory markers in the mild stages of disease.^6^ Furthermore, genome-wide association studies (GWAS) have identified several risk AD candidate genes for inflammatory pathways, which are strongly supportive of inflammation playing a critical role in early AD aetiology.^7^

Dementia with Lewy bodies (DLB) is the second most common cause of degenerative dementia, and shares many clinical characteristics with AD. A recent meta-analysis showed that there is increased systemic inflammation in patients with Parkinson’s disease, which is closely related to DLB.^8^ It is therefore likely that the neuroinflammatory processes occurring in AD are also involved in driving neurodegeneration in DLB, with cytokines such as interleukin (IL)-1 being implicated in the neuropathological changes characteristic of both conditions.^9^

Only two studies have assessed inflammation in patients with DLB, both using cerebrospinal fluid (CSF), with one finding lower levels of IL-6 and the other finding no significant difference.^10 11^ No studies have examined inflammatory markers in the blood compared with controls, although a small exploratory study found that increased peripheral levels of IL-6 were associated with cognitive impairment in patients with DLB.^12^ The accessibility and practicality of using peripheral blood to monitor neuroinflammation in patients make this an attractive option. Furthermore, the increasing evidence that peripheral inflammation and neuroinflammation are closely related suggests that altered systemic inflammatory markers reflect neurodegenerative disease.^13^ Therefore, there is a need to investigate systemic inflammatory markers in patients with DLB as markers of brain inflammation.

The aim of this study was to characterise the plasma cytokine profile and C-reactive protein (CRP) levels of patients with DLB and compare this with patients with AD and healthy control subjects. Furthermore, we aimed to compare this with patients with mild cognitive impairment (MCI) thought to be due to AD and DLB to assess inflammation at this earlier pre-dementia stage. We hypothesised that the inflammatory profile in patients with DLB would be similar to that seen in patients with AD, and would be raised compared with healthy control subjects. Furthermore, we hypothesised that patients with MCI would have raised inflammatory markers compared with healthy control subjects and patients with dementia.

## Methods

### Subjects

Patients with dementia and MCI were recruited from the same memory clinics and dementia services including neurology and geriatrics in the North-East of England. All patients were aged over 60 and had provided written informed consent, or in cases where capacity was lacking their participation in the study was discussed with a consultee in accordance with UK legislation.

Patients with dementia had a Mini Mental State Examination (MMSE) score of at least 12. Diagnosis of DLB was made based on the International Consensus criteria,^14^ and patients with AD were diagnosed based on the National Institute of Neurological and Communicative Disorders and Stroke and the Alzheimer’s Disease and Related Disorders Association criteria.^15 16^ Patients with MCI met the National Institute on Aging and Alzheimer’s Association MCI criteria.^16^ Probable Lewy Body MCI (MCI-LB) was diagnosed using the Diagnostic and Statistical Manual of Mental Disorders 5 criteria,^17^ with the inclusion of dopaminergic imaging so that probable MCI-LB was diagnosed when at least two core or suggestive features of DLB were present, but in the absence of dementia. If none of these symptoms were present, the patient was classified as MCI-AD. Dementia and MCI diagnoses were made by a consensus panel of three experienced clinicians. Healthy control subjects were recruited from patients’ friends or family, or people who had already indicated a willingness to participate in research.

We further classified participants as having amnestic subtype if their Rey delay score was less than 3 and as non-amnestic if their Rey delay score was 3 or more in order to ascertain whether there was any difference in inflammation.

Exclusion criteria included severe physical, neurological or psychiatric illness, history of alcohol excess, and use of psychotropic drugs. We also excluded people who had a history or evidence of a stroke and those with possible or probable vascular dementia. We did not exclude people with inflammatory or autoimmune diseases; however, information regarding this was collected and taken into account during analysis.

### Assessments

All patients were assessed by the equivalent of a board-certified medical practitioner, including a physical and neurological examination, and assessment of parkinsonism used the Movement Disorder Society Unified Parkinson’s Disease Rating Scale Part III (UPDRS). They were free from acute inflammatory illness, and their illness burden was assessed using the Cumulative Illness Rating Scale-Geriatrics (CIRS-G). Cognition was assessed using the Addenbrooke’s Cognitive Examination Revised (ACE-R) and MMSE.

Psychiatric symptoms were assessed using the Neuropsychiatric Inventory and the Geriatric Depression Scale. Activities of daily living were assessed using the Instrumental Activities of Daily Living Scale and Bristol Activities of Daily Living Scale. Doses of levodopa and antidementia medications were documented. Based on these assessments, healthy control subjects did not show any evidence of dementia. Positron emission tomography (PET) imaging was carried out on all dementia and healthy comparison subjects to investigate the amyloid burden in the brain.

### Measurement of cytokines and high-sensitivity CRP

Venous blood samples were taken from all subjects using EDTA tubes, which were then centrifuged and the plasma removed. Samples were stored at −80° until assays were performed. Cytokine assays were performed using the Meso Scale Discovery V-Plex Plus Proinflammatory Panel 1, which included interferon (IFN)-gamma, IL-1beta, IL-2, IL-4, IL-6, IL-8, IL-10, IL-12p70, IL-13 and TNF-alpha. Assays were analysed at the National Institute for Health and Research-Newcastle University Biomarkers Facility according to the manufacturer’s protocol, and samples were processed in triplicates. Samples that were under the limit of detection for a particular cytokine (<0.05 for IFN-gamma, <0.03 for IL-13, <0.02 for IL-12p70 and <0.01 for all other cytokines) had cytokine levels that were low enough to be undistinguishable from background noise, and therefore these samples were treated as having ‘zero’ levels of that cytokine. High-sensitivity CRP (hsCRP) was analysed using Roche cobas c702, and was carried out at the Royal Victoria Infirmary in Newcastle upon Tyne.

### Analysis

Statistical analysis was completed using IBM SPSS Statistics V.23 software (http://www-03.ibm.com/software/products/en/spss-statistics). Data distribution and normality were assessed using the Shapiro-Wilk test, and log transformations were performed to attempt to normalise any skewed data. Comparisons between diagnostic groups were carried out using analysis of variance or Kruskal-Wallis tests depending on normality of the data following log transformations. Where gender differences between groups were present, gender was added as a covariant in the analysis. Bonferroni correction was used to correct for multiple comparisons, so that P<0.001 was required for significance. Correlations were sought between inflammatory markers and patient characteristics using Spearman’s rank correlation, due to some of the data not being normally distributed following log transformation. Finally, significant differences in cytokine levels were sought between participants with amyloid-positive and amyloid-negative PET scans, and between participants with amnestic and non-amnestic subtype.

## Results

One hundred and thirty-six subjects took part in this study. Of these, 20 were healthy control subjects, 20 with AD, 37 with DLB, 38 with MCI-LB and 21 with MCI-AD (see table 1 for full details of subject characteristics). The mean age of the whole cohort was 76.2 (±6.9) years, and this was not significantly different between any of the groups. In all groups except the MCI-AD group, there were more men than women. Disease duration was around 2 years in both the DLB and AD patients. As expected, UPDRS was higher in the DLB groups compared with all other groups, the ACE-R and MMSE scores were lower in the dementia and MCI groups compared with healthy control subjects, and a higher proportion of patients with dementia were taking anti-dementia medications than patients with MCI. The proportion of patients taking anti-inflammatory medications ranged between 25% and 66%, but in the majority of cases this was low-dose aspirin (75 mg), prescribed for its vascular effects. Eight participants were taking a non-aspirin non-steroidal anti-inflammatory drug, and seven were taking a steroid; these were spread evenly between all groups.

**Table 1:** Differential diagnosis for decreased conscious level

	Controls (n=20)	DLB (n=37)	AD (n=20)	MCI-LB (n=38)	MCI-AD (n=21)
Age (years±SD)	75.9±7.3	76.1±6.6	75.9±6.7	75.6±7.5	78.5±6.4
Sex (M:F)	16:4	30:7§	15:5	25:13	7:14*
Disease duration (months±SD)		25.2±20.4	24.2±20.9		
MMSE (score±SD)	29.1±0.9*†‡§	21.2±4.6‡§¶	20.3±4.7‡§¶	26.4±2.3*†¶	26.5±2.1*†¶
ACE (score±SD)	94.8±3.0*†‡§	62.1±15.4‡§¶	60.5±16.5‡§¶	78.2±9.6*†¶	79.4±11.1*†¶
UPDRS (score±SD)	5.6±3.5*†‡§	43.0±18.1†‡§¶	13.6±7.2*‡¶	27.2±16.2*†§¶	15.7±6.5*‡¶
NPI (score±SD)		20.4±19.9§	14.0±13.3§	14.3±10.5§	5.1±6.2*†‡
Levodopa use (no of patients, %)	0 (0%)	13 (35%)	0 (0%)	8 (21%)	0 (0%)
Antidementia medication (no of patients, %)	–	35 (96%)‡§	20 (100%)‡§	18 (47%)*†	5 (24%)*†
Anti-inflammatory medication (no of patients, %)	13 (65%)	14 (38%)	5 (25%)	23 (66%)	9 (43%)
CIRS-G (score±SD)	6.7±4.3*	11.5±4.1¶	8.6±3.6	9.5±4.3	9.5±3.9
CIRS-G without neuro/psych (score±SD)	6.5±4.1	8.1±3.8	6.2±3.4	8.4±4.5	8.2±4.4

Results are presented as mean and SD.

Antidementia medication includes donepezil, rivastigmine, galantamine and memantine. Anti-inflammatory medication includes all non-steroidal anti-inflammatory drugs and steroids.

*Significantly different from DLB group (P<0.05).

†Significantly different from AD group (P<0.05)

‡Significantly different from MCI-LB group (P<0.05)

§Significantly different from MCI-AD group (P<0.05)

¶Significantly different from controls (P<0.05).

ACE, Addenbrooke’s Cognitive Examination; AD, Alzheimer’s Disease; CIRS-G, Cumulative Illness Rating Scale-Geriatric; CIRS-G without neuro/psych, CIRS-G score without the neurology or psychiatric components included; DLB, dementia with Lewy bodies; MCI, mild cognitive impairment; MCI-AD, MCI with probable Alzheimer’s disease; MCI-LB, MCI with probable Lewy body dementia; MMSE, Mini Mental State Examination; NPI, Neuropsychiatric Inventory; UPDRS, Unified Parkinson’s Disease Rating Scale.

The CIRS-G identified that the DLB group had a significantly higher general medical illness score than other groups. However, this was due to the DLB group scoring more highly in the neurological and psychiatric aspects of the assessment, as would be expected by the increased incidence of parkinsonism and hallucinations in these groups of patients. Once these neurological and psychological aspects had been removed from analysis, there were no significant differences between any of the groups (see table 1).

We also looked specifically at the prevalence of autoimmune comorbidities in each group due to the impact it can have on inflammation. Overall one control participant had rheumatoid arthritis, one or two participants in each group had hypothyroidism or hyperthyroidism, one control participant had giant cell arteritis, two patients with DLB had polymyalgia, one patient with DLB and one patient with MCI-LB had gout, and one control participant had ‘immune system disease’ not otherwise specified. Therefore the prevalence of autoimmune inflammatory disease was spread evenly across groups. Overall all other comorbidities including vascular comorbidities were spread evenly between groups.

### Inflammatory marker levels

Table 2 shows all cytokine and hsCRP results. Overall, patients in both MCI groups had significantly higher levels of IL-10, IL-1beta, IL-4 and IL-2 than control, AD or DLB groups. Both MCI groups had lower levels of TNF-alpha than the control or dementia groups. IL-13 was removed because more than 50% of the samples had non-detectable cytokine levels. Furthermore, IL-12p70 was removed due to the high interassay variability. There were no significant differences between either of the dementia groups and control subjects, and there were no significant differences between the two dementia groups or between the two MCI groups (table 2). Cohen’s effect sizes were calculated and were large (d>0.8) for all significant differences, apart from that between the AD group and MCI groups for IL-10, which were moderate (d=0.55 and d=0.56 for AD vs MCI-LB and AD vs MCI-AD respectively). The median values for intra-assay and interassay coefficient of variance across all cytokines were 9.87% and 16.04%, respectively. Correction calculations were carried out based on the values obtained for internal controls to account for this variation. There were no significant differences in hsCRP level between any of the groups (table 2).

**Table 2:** Level of inflammatory markers in patients and controls

	Control	DLB	AD	MCI-LB	MCI-AD
CRP, mg/mL	3.35±5.72	1.73±2.12	6.29±14.56	4.12±6.44	3.41±3.34
IFN-gamma, pg/L	7.56±6.13	5.13±4.13	9.01±12.06	6.52±4.30	8.75±12.08
IL-10, pg/L	0.46±0.62	0.43±0.52	0.60±0.75	0.96±0.54*†‡	0.93±0.36*†‡
IL-1beta, pg/L	0.51±2.16	0.07±0.33	0.03±0.08	1.92±1.24*†‡	2.72±1.51*†‡
IL-2, pg/L	0.38±0.42	0.34±0.37	0.39±0.68	3.68±0.85*†‡	3.56±0.92*†‡
IL-4, pg/L	0.03±0.05	0.02±0.03	0.01±0.03	0.80±0.16*†‡	0.85±0.21*†‡
IL-6, pg/l	1.66±0.99	2.02±2.00	2.78±2.21	2.90±10.39	3.32±4.34
IL-8, pg/L	4.85±1.49	5.10±2.03	5.11±2.25	6.75±17.50	4.34±2.88
TNF-alpha, pg/L	4.26±1.32	4.02±1.61	4.28±2.38	2.23±2.01*†‡	3.08±3.53*†

Results are presented as mean±SD.

*Significantly different from controls (P≤0.001).

†Significantly different from DLB group (P≤0.001).

‡Significantly different from AD group (P≤0.001).

AD, Alzheimer’s disease; CRP, C reactive protein; DLB, dementia with Lewy bodies; IFN-gamma, interferon-gamma; IL, interleukin; MCI, mild cognitive impairment; MCI-AD, MCI with probable Alzheimer’s disease; MCI-LB, MCI with probable Lewy body dementia; TNF-alpha, tumour necrosis factor-alpha.

### Correlations

**Figure 1:**
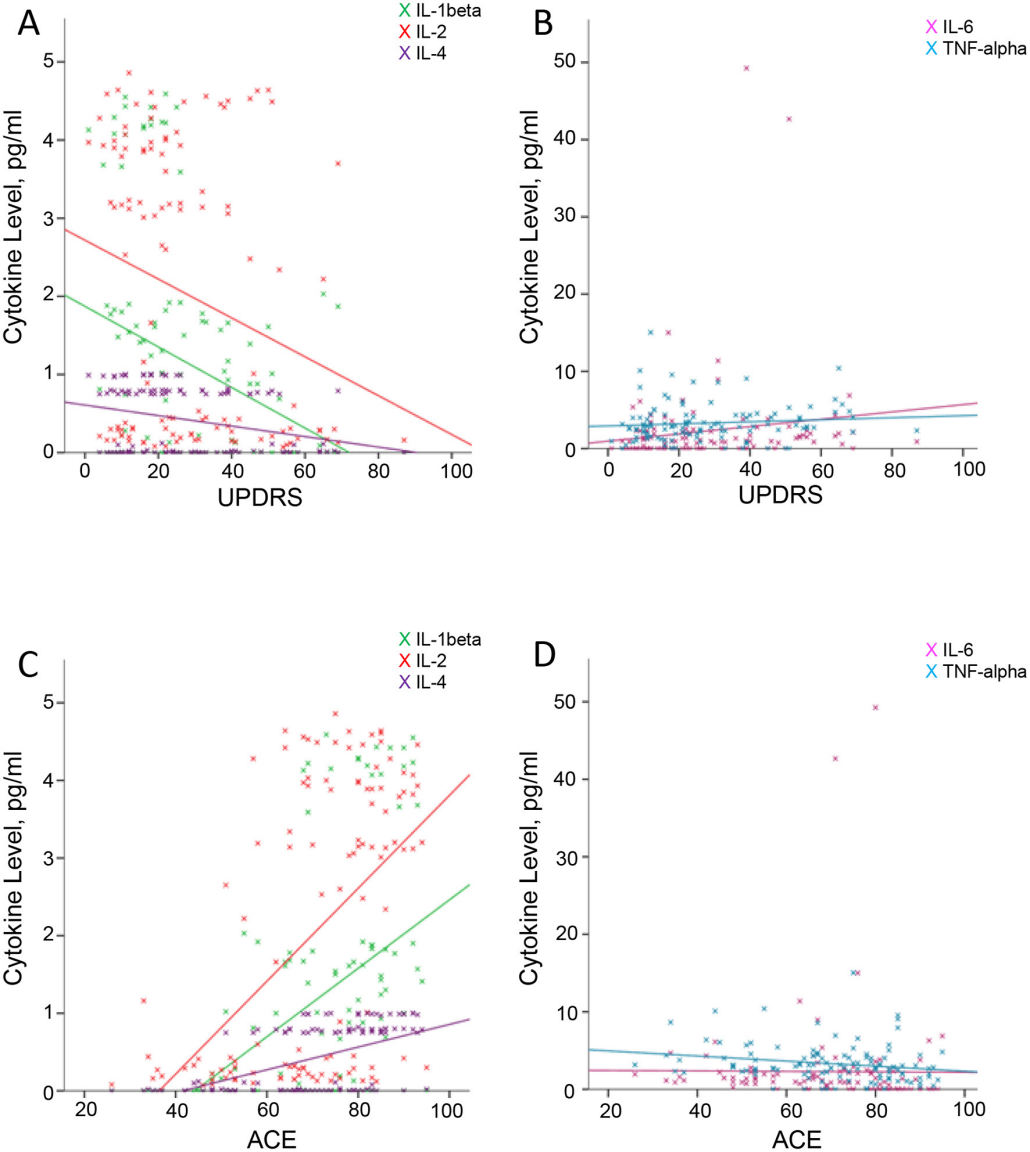
Correlations between Unified Parkinson’s Disease Rating Scale (UPDRS) and Addenbrooke’s Cognitive Examination (ACE) scores with cytokine levels. (A,B) Correlations between UPDRS and interleukin (IL)-1beta (r=−0.285, P=0.002), IL-2 (r=−0.220, P=0.018) and IL-4 (r=−0.217, P=0.019), and between UPDRS and IL-6 (r=0.208, P=0.025) and tumour necrosis factor (TNF)-alpha (r=0.168, P=0.071). (C,D) Correlations between the ACE score and IL-1beta (r=0.494, P=0.000), IL-2 (r=0.485, P=0.000) and IL-4 (r=0.540, P=0.000), and between the ACE and IL-6 (r=−0.343, P=0.000) and TNF-alpha (r=−0.253, P=0.006).

Spearman’s rank correlation was used to explore any correlations between inflammatory markers and patient characteristics. Healthy control subjects were excluded from the correlation analysis. Increasing age was correlated with a higher level of IFN-gamma. Greater severity of cognitive impairment measured using the ACE-R and the MMSE was associated with having a significantly lower level of IL-1beta, IL-2 and IL-4, and a higher level of IL-6 and TNF-alpha (figure 1).

Correlations were also sought between the level of parkinsonism and inflammatory markers using the UPDRS. AD groups were also removed from this analysis, as parkinsonism is not a feature in this group and scores were consequently low. Overall, a significantly higher score on the UPDRS was associated with having lower levels of IL-1beta, IL-2 and IL-4, and a higher level of IL-6 and TNF-alpha.

When looking at the MCI and dementia groups separately, a greater level of parkinsonism was associated with a lower level of IL-1beta and IL-4 in the MCI-LB group only. In the DLB group, the only significant correlation was between having more severe parkinsonian symptoms and a higher level of IL-10.

### Inflammatory markers and amyloid

There were no significant differences in any of the cytokine levels between participants with amyloid-positive and amyloid-negative PET scans.

### Inflammatory marker in amnesic versus non-amnesic patients

There were no significant differences in any of the cytokine levels between participants with amnestic and non-amnestic MCI subtype.

## Discussion

We investigated for the first time the peripheral cytokine profile in DLB and AD both at the MCI stage and at the dementia stage, and compared with healthy control subjects. We found that while there was no difference in the level of inflammatory markers between control and dementia subjects, there was a significant increase in inflammatory markers at the MCI stage. Specifically, we found significantly higher levels of IL-10, IL-1beta, IL-4 and IL-2 in both MCI-LB and MCI-AD compared with dementia and control subjects. Furthermore, greater disease severity, whether measured for cognition or parkinsonism, was associated with lower levels of IL-1beta, IL-2 and IL-4, further supporting our finding that inflammatory markers decrease with disease progression. To our knowledge this is the first study to investigate baseline inflammatory markers in DLB with control subjects, and we found that peripheral inflammation in DLB shows a similar pattern to that seen in AD.

The results from this study are consistent with previous studies of AD. Previous studies have demonstrated lower levels of IL-1beta, Il-6, IL-12, IL-16, IL-18 and TGF-β1 in severe AD compared with mild or moderate disease.^6 18^ CSF chemokine levels have also been found to be increased in MCI compared with patients with AD.^19^ Peripheral blood mononuclear cell stimulation studies have found similar patterns of immune dysfunction; one study found higher levels of cytokine production in patients with MCI than AD or controls,^20^ and another found significantly lower levels of cytokine release in severe AD.^18 21^ A recent meta-analysis found that increased peripheral levels of inflammatory markers are associated with an increased risk of later dementia, indicating that peripheral inflammation may occur before clinical symptoms are present.^22^

Few studies have investigated inflammatory markers in DLB. One study found that IL-6 in CSF was negatively correlated with MMSE,^11^ and a correlation study found that increasing IL-6 is associated with cognitive decline.^12^ In our study IL-6 was one of the only cytokines that were negatively correlated with cognitive function, and it was also correlated with worsening parkinsonian symptoms. However, studies investigating Parkinson’s disease suggest that high IL-6 levels increase the risk of later Parkinson’s disease, implying that high IL-6 may be associated with triggering disease onset.^23^ These findings suggest that IL-6 may show a more complex relationship with disease progression, perhaps increasing both early and late in disease.

The only cytokine that we found to be decreased in patients with MCI was TNF-alpha, which was also associated with worsening cognition and parkinsonism. Previous studies investigating TNF-alpha in AD have found mixed results. While some studies have found increased levels of TNF-alpha in patients with AD,^24 25^ some have found decreased levels^26 27^ and others have found no difference.^28^ Studies investigating TNF-alpha levels at different stages of disease have in general found higher levels of TNF-alpha in patients with severe AD compared with mild AD and MCI,^29 30^ and therefore it may be that the increase in TNF-alpha is seen at a later stage in disease than other cytokines.

The underlying cause for this inflammation seen early in disease is still not fully understood, although it has been suggested that in the early stages of disease, aberrant protein deposition induces a microglial inflammatory response in the brain, leading to peripheral inflammation.^31^ It has been suggested that it is this initial microglial activation that produces toxic products, producing further neuronal death and thereby perpetuating the inflammatory reaction.^32^ Previously, studies have also found increased inflammation peripherally in Parkinson’s disease,^8 33^ suggesting that inflammation may indeed be associated with aberrant protein aggregation and not specifically beta amyloid (Aβ). However studies investigating inflammation in Parkinson’s disease dementia have found mixed results,^34 35^ which could perhaps be a reflection of the severity of disease of this cohort of participants, with such patients being less likely to be in the early stages of disease.

Although it is not clear how systemic inflammation relates to disease processes occurring in the brain, studies suggest that peripheral inflammation and central inflammation are closely related.^2^ Microglia expressing IL-1 appear to be more highly associated with early-stage than late-stage Aβ plaques in AD brains, and IL-1 may be important in driving plaque progression.^36^ Furthermore, administration of Aβ centrally in mice induces a dose-dependent increase in peripheral IL-6,^37^ suggesting that there is some relationship between peripheral inflammatory markers and central disease progression. It has been suggested that Aβ deposition may drive cognitive decline in people with AD, DLB and PD, perhaps by inducing inflammation; however, evidence is limited and results are inconsistent.^38^ The present study found no relationship between Aβ and inflammatory marker levels, suggesting that inflammation may not be a direct consequence of Aβ deposition. A previous study showed that inflammation appears to be a better marker for synapse loss seen in AD than Aβ deposition, suggesting that there is a role for inflammation driving neurodegeneration.^39^ This study suggests that while there is a clear increase in inflammation at the MCI stage, there appears to be an imbalance between proinflammatory and anti-inflammatory cytokines, something that is thought to be highly important in the progression from acute inflammation to chronic disease.^40^

This study did not investigate patients with severe dementia; however, the correlation analysis suggests that as disease progresses, the level of inflammation decreases. Previous studies investigating this have indeed shown that there appears to be a gradual decrease in the level of inflammatory markers with disease progression, reaching levels below healthy in severe disease.^6 18^ However, although chronic inflammation appears to decrease with disease severity, acute inflammation still likely has an impact on the rate of decline, as it has been shown that acute increases in TNF-alpha in patients with dementia predict more rapid disease progression.^2^

This decrease in chronic inflammation seen later in disease may relate to the chronic neurodegenerative processes involved in dementia progression. It has been shown in transgenic mice that chronic Aβ exposure in the brain is associated with a decreased immune response.^41^ It may be that chronic exposure to Aβ in AD leads to increased tolerance for Aβ deposition. This may result in immune hyporesponsiveness, which may contribute towards further Aβ deposition. It seems likely that a similar inflammatory process is occurring in DLB; however, more evidence is needed to ascertain whether this is also related to beta deposition, or whether this is as a result of alpha-synuclein. Therefore, it could be hypothesised that while aberrant protein deposition in the brain in AD and DLB may initially trigger an immune response leading to an increase in inflammatory markers and therefore neuronal toxicity, chronic exposure may lead to tolerance and a hyporesponsive immune system.

These findings have important implications for the management of dementia. Previous studies have investigated the use of anti-inflammatory medication in the treatment of AD and found no clinical benefit.^42^ However, because the patients in these cohorts had already progressed from MCI to AD, it is likely that the stage of dementia was too advanced for the anti-inflammatory medication to have had any clinical benefit. Observational studies have found that long-term use of anti-inflammatory medications is associated with a reduced risk of dementia, indicating that the beneficial effects of anti-inflammatory medications may only apply in very early stages of disease.^43^ Further studies are needed to ascertain the optimum time, dose and type of anti-inflammatory medication to be used in the management of patients with MCI. More recent studies have suggested that there may be a role for more specific anti-inflammatory agents; a pilot study found improvements in cognition in patients with AD taking specific TNF-alpha inhibitors.^44^ Randomised controlled trials are needed to ascertain the benefit of anti-inflammatory antibody therapies in AD and DLB.

Strengths of this study include the large patient group used and the thorough clinical assessment of each patient by an experienced clinician using detailed rating scales. Robust exclusion criteria were used, including routine blood tests to assess for systemic disease. Limitations include the cross-sectional design of the study, the high number of samples falling below the detection level for IL-13 leading to exclusion of IL-13, and the severity of the dementia groups, having excluded patients with an MMSE score of less than 12.

In conclusion, we have shown for the first time that in both AD and DLB, increased peripheral inflammation occurs early at the MCI stage and decreases with severity of disease. It seems likely therefore that there is an early microglial response to aberrant protein deposition, which progresses to an immune tolerance in chronic disease leading to immune hyporesponsiveness. Early treatment of AD and DLB at the MCI stage with anti-inflammatory medication may therefore be of benefit, and our results indicate that further trials should focus on early-stage disease.
